# Point-of-care HIV tests done by peers, Brazil

**DOI:** 10.2471/BLT.15.162461

**Published:** 2016-06-02

**Authors:** Ana Roberta Pati Pascom, Clarissa Habckost Dutra de Barros, Tainah Dourado de Miranda Lobo, Elisiane Nelcina Pasini, Regina Aparecida Comparini, Fábio Caldas de Mesquita

**Affiliations:** a STI, AIDS and Viral Hepatitis Department, Health Surveillance Secretariat, Ministry of Health, Setor Administrativo Federal Sul (SAFS) 02 – Bloco F – Ed. Premium Torre I, Sala 4, 70.070–600, Brasília, DF, Brazil.

## Abstract

**Problem:**

Early diagnosis of infections with human immunodeficiency virus (HIV) is needed – especially among key populations such as sex workers, transgender people, men who have sex with men and people who use drugs.

**Approach:**

The Brazilian Ministry of Health developed a strategy called *Viva Melhor Sabendo* (“live better knowing”) to increase HIV testing among key populations. In partnership with nongovernmental organizations (NGOs), a peer point-of-care testing intervention, using an oral fluid rapid test, was introduced at social venues for key populations at different times of the day.

**Local setting:**

Key populations in Brazil can have 40 times higher HIV prevalence than the general population (14.8% versus 0.4%).

**Relevant changes:**

Legislation was reinterpreted, so that oral fluid rapid tests could be administered by any person trained in rapid testing by the health ministry. Between January 2014 and March 2015, 29 723 oral fluid tests were administered; 791 (2.7%) were positive. Among the key populations, transgender people had the greatest proportion of positive results (10.7%; 172/1612), followed by men who declared themselves as commercial sex workers (8.7%; 165/1889) and men who have sex with men (4.8%; 292/6055).

**Lessons learnt:**

The strategy improved access to HIV testing. Testing done by peers at times and locations suitable for key populations increased acceptance of testing. Working with relevant NGOs is a useful approach when reaching out to these key populations.

## Résumé

**Problème:**

Il est nécessaire de procéder à un diagnostic précoce des infections par le virus de l'immunodéficience humaine (VIH), en particulier chez les groupes de population clés que forment notamment les travailleurs du sexe, les transsexuels, les hommes ayant des rapports sexuels avec des hommes et les personnes qui consomment des drogues.

**Approche:**

Le ministère brésilien de la Santé a mis au point une stratégie appelée *Viva Melhor Sabendo* (« Vivre mieux en étant informé ») pour accroître le dépistage du VIH dans les groupes de population clés. En partenariat avec des organisations non gouvernementales (ONG), des actions de dépistage par des pairs sur le lieu des soins, à l'aide d'un test salivaire rapide, ont été mises en place à différents moments de la journée dans des établissements sociaux pour les groupes de population clés.

**Environnement local:**

Au Brésil, la prévalence de l'infection par le VIH peut être jusqu'à 40 fois plus élevée dans les groupes de population clés que dans la population générale (14,8% contre 0,4%).

**Changements significatifs:**

La législation a été révisée de façon à ce que les tests salivaires rapides puissent être réalisés par toute personne formée aux méthodes de dépistage rapide par le ministère de la Santé. Sur les 29 723 tests salivaires effectués entre janvier 2014 et mars 2015, 791 (2,7%) étaient positifs. Dans les groupes de population clés, la plus forte proportion de résultats positifs revenait aux transsexuels (10,7%; 172/1612), suivis par les hommes qui se définissent eux même comme étant des travailleurs de l'industrie du sexe (8,7%; 165/1889) et les hommes ayant des rapports sexuels avec des hommes (4,8%; 292/6055).

**Leçons tirées:**

La stratégie a permis d'améliorer l'accès au dépistage du VIH. La réalisation de tests par des pairs à des moments et dans des endroits appropriés pour les groupes de population clés a favorisé l'acceptation du dépistage. Il est utile de travailler avec les ONG compétentes pour approcher ces groupes de population clés.

## Resumen

**Situación:**

Es necesario un diagnóstico temprano de infecciones por el virus de la inmunodeficiencia humana (VIH), especialmente en poblaciones clave como trabajadores sexuales, personas transexuales, hombres que mantienen relaciones sexuales con otros hombres y consumidores de drogas.

**Enfoque:**

El Ministerio de Sanidad brasileño desarrolló una estrategia denominada *Viva Melhor Sabendo*****(“vive mejor sabiendo”) para aumentar las pruebas del VIH en poblaciones clave. En asociación con organizaciones no gubernamentales (ONG), se introdujo una intervención de prueba en el punto de atención realizada por expertos, mediante la utilización de una prueba rápida de fluidos orales, en centros sociales para poblaciones clave en distintos momentos del día.

**Marco regional:**

Las poblaciones clave en Brasil pueden tener una prevalencia de VIH 40 veces mayor que la población general (un 14,8% frente a un 0,4%).

**Cambios importantes:**

Se reinterpretó la legislación, de modo que cualquier persona formada en pruebas rápidas por el Ministerio de Sanidad pueda administrar las pruebas rápidas de fluidos orales. Entre enero de 2014 y marzo de 2015, se administraron 29 723 pruebas de fluidos orales, de las cuales 791 (2,7%) dieron resultados positivos. Entre las poblaciones clave, las personas transexuales tuvieron el mayor porcentaje de resultados positivos (10,7%; 172/1 612), seguidas de los hombres que declararon ser trabajadores sexuales (8,7%; 165/1 889) y los hombres que mantienen relaciones sexuales con otros hombres (4,8%; 292/6 055).

**Lecciones aprendidas:**

La estrategia mejoró el acceso a las pruebas del VIH. Las pruebas realizadas por expertos en momentos y lugares adecuados para poblaciones clave aumentaron la aceptación de las pruebas. Trabajar con ONG importantes es un enfoque útil a la hora de dirigirse a dichas poblaciones clave.

## ملخص

**المشكلة:**

يلزم ****إجراء التشخيص المبكر لحالات الإصابة بفيروس عوز المناعة البشري (HIV)، وخاصةً في أوساط القطاعات السكانية الرئيسة، مثل المشتغلين بالجنس، والمتحولين جنسيًا، والرجال الذين باشروا العلاقة الحميمة مع رجال آخرين، ومتعاطي المخدرات.

**الأسلوب:**

قامت وزارة الصحة البرازيلية بوضع استراتيجية حملت اسم *Viva Melhor Sabendo * (أي "تمتع بحياة أفضل في ظل المعرفة") لزيادة إجراء اختبارات الكشف عن الإصابة بعوز المناعة البشري في أوساط القطاعات السكانية الرئيسة. وفي إطار الشراكة مع المنظمات غير الحكومية، فقد تم طرح برنامج للتدخل بإجراء الفحوصات على أيدي الأقران في أماكن الرعاية الصحية باستخدام اختبار سريع النتائج للسائل الفموي وذلك في دور الخدمات الاجتماعات للقطاعات السكانية الرئيسة في أوقات مختلفة من اليوم.

**المواقع المحلية:**

يمكن للقطاعات السكانية الرئيسة في البرازيل أن تواجه نسبة أكبر 40 مرة لشيوع الإصابة بفيروس عوز المناعة البشري بالمقارنة مع عموم السكان (بواقع 14.8‏% في مقابل 0.4‏%).

**التغييرات ذات الصلة:**

تمت إعادة تأويل التشريعات القانونية بما يسمح بإجراء الاختبارات سريعة النتائج للسائل الفموي على أيدي أي شخص تلقى تدريبه لدى وزارة الصحة على إجراء الاختبارات السريعة. وشهدت الفترة الممتدة ما بين شهر يناير/كانون الثاني من عام 2014 وحتى مارس/آذار من عام 2015 إجراء 29,723 اختبار للسائل الفموي، خرجت 791 بنتائج إيجابية (أي بنسبة 2.7‏%). ومن بين القطاعات السكانية الرئيسة، فقط ظهرت بين المتحولين جنسيًا أعلى نسبة من النتائج الإيجابية (بنسبة 10.7‏%، بواقع 172 من إجمالي 1612)، يليهم الرجال الذين صرّحوا بأنهم من المشتغلين بالجنس لأغراض تجارية (بنسبة بلغت 8.7‏%، بواقع 165 من إجمالي 1889)، ثم الرجال الذين عاشروا رجالاً آخرين (بنسبة 4.8‏% بواقع 292 من إجمالي 6055).

**الدروس المستفادة:**

أدت الاستراتيجية إلى تحسين سبل الاستفادة من اختبارات الكشف عن عوز المناعة البشري، فقد زادت الاختبارات التي أجراها الأقران في الأوقات والأماكن المناسبة للقطاعات السكانية الرئيسة من نسبة القبول تجاه الخضوع للاختبارات. كما أن التعاون مع المنظمات غير الحكومية يمثل أسلوبًا مفيدًا عند الوصول إلى تلك القطاعات السكانية الرئيسة.

## 摘要

**问题:**

我们急需开展人体免疫缺陷病毒 (HIV) 感染的早期诊断——尤其是在性工作者、跨性别者、进行同性性行为的男同性恋者以及吸毒人群等重点人群中。

**方法:**

巴西卫生部开发了一种名为 *Viva Melhor Sabendo* （“了解让生活更美好”）的方案以提高重点人群中的 HIV 检测率。 通过与非政府组织 (NGO) 的合作，开发出一项可利用口腔液体快速检测的即时检测干预，可在一天内不同时段在重点人群的社交场合开展。

**当地环境:**

巴西重点人群中的 HIV 感染率可达到普通人群 HIV 感染率的 40 倍（14.8% 比 0.4%）。

**相关改变:**

对法律进行重新解释，因此，任何经卫生部快速检测培训的人员都可执行口腔液体检测。 在 2014 年 1 月至 2015 年 3 月之间，一共进行了 29 723 项口腔液体检测，其中 791 (2.7%) 例呈阳性。 在重点人群中，跨性别者的阳性检测结果所占比例最高 (10.7%; 172/1612)，其次是自称为性工作者的男性 (8.7%; 165/1889) 以及进行同性性行为的男同性恋者 (4.8%; 292/6055)。

**经验教训:**

该项措施提高了 HIV 的检测率。 在适合重点人群的地点和时间，由同伴进行的检测提高了检测的接受度。 与相关 NGO 合作对于覆盖上述重点人群是一个有效方法。

## Резюме

**Проблема:**

Раннее диагностирование заражений вирусом иммунодефицита человека (ВИЧ) необходимо, особенно среди представителей ключевых групп населения, к которым относятся работники секс-индустрии, транссексуалы, мужчины, практикующие секс с мужчинами, и люди, употребляющие наркотики.

**Подход:**

Министерством здравоохранения Бразилии была разработана стратегия под названием Viva Melhor Sabendo (Жить лучше зная), нацеленная на распространение тестирования на ВИЧ среди ключевых групп населения. В партнерстве с неправительственными организациями (НПО) тестирование по месту лечения, проводимое людьми из одной и той же группы населения, в котором использовался экспресс-анализ ротовой жидкости, было представлено для ключевых групп населения в местах проведения общественных мероприятий в разное время суток.

**Местные условия:**

Показатели распространенности ВИЧ среди представителей ключевых групп населения в Бразилии могут в 40 раз превышать показатели распространенности этого вируса среди населения в целом (14,8% против 0,4%).

**Осуществленные перемены:**

Законодательство было пересмотрено так, чтобы экспресс-анализы ротовой жидкости мог выполнить любой человек, которого Министерство здравоохранения обучило выполнению соответствующего экспресс-анализа. За период с января 2014 года по март 2015 года было выполнено 29 723 теста ротовой жидкости, результаты 791 (2,7%) из них были положительными. Среди представителей ключевых групп населения наибольшая доля положительных результатов пришлась на транссексуалов (10,7%; 172 из 1612); меньшие доли пришлись на людей, которые заявили, что являются работниками сексуальной индустрии (8,7%; 165 из 1889), и мужчин, практикующих секс с мужчинами (4,8%; 292 из 6055).

**Выводы:**

В результате применения стратегии увеличилась доступность тестирования на ВИЧ. Согласие на тестирование стало более частым благодаря тому, что оно выполнялось людьми из той же группы населения, что и тестируемый, в такое время и в таком месте, которые удобны для представителей ключевых групп населения. Сотрудничество с соответствующими НПО является целесообразным подходом, когда необходимо оказать помощь этим ключевым группам населения.

## Introduction

To respond to the human immunodeficiency virus (HIV) epidemic it is important to diagnose HIV-infected people early, because diagnosis allows infected people to start antiretroviral therapy (ART) and – by reducing their viral load – reduces HIV transmission.^1^ However, fear of the disease and the consequences of being infected make people hesitant to test themselves for HIV.^2^^–^^4^

Key populations –men who have sex with men, transgender people, sex workers and people who use drugs – are disproportionally affected by the HIV epidemic.^5^ Their risk of infection is higher than the general population and they face legal and social barriers in accessing health services. To improve access to HIV care and diagnosis, community-based interventions are essential.^6^^,^^7^

To expand HIV testing for key populations in Brazil, the health ministry developed a community-based testing strategy, called *Viva Melhor Sabendo* (“live better knowing”). We present lessons learnt during the first 15 months of its implementation.

## Local context

In Brazil, the HIV epidemic is largely concentrated in key populations. For men who have sex with men, prevalence was 14.8% in 2015, 40 times higher than in the general population (0.4% in 2012). In other key populations the prevalence is about 5% (female commercial sex workers 4.9% in 2009; drug users 5.0% in 2013).^8^^–^^12^

The Brazilian acquired immunodeficiency syndrome (AIDS) programme has a set of preventive actions for key populations such as financial support for specific activities, distribution of educational and prevention materials – such as male and female condoms – and workshops on HIV prevention. These actions are done in partnership with nongovernmental organizations (NGOs). Since 2013, the health ministry aims to provide ART to every person living with HIV, irrespective of their CD4+ T-lymphocyte count.^13^

## Approach

Once the programme implemented treatment as prevention, early diagnosis emerged as the next requirement, especially for key populations. To meet this need, the Department of Sexually Transmitted Infections (STIs), AIDS and Viral Hepatitis developed a key populations-focused strategy, which combined prevention, testing and counselling initiatives. The strategy included a peer point-of-care testing intervention with an oral fluid HIV rapid test. The test, DPP® HIV-1/2 (Biomanguinhos/Fiocruz, Rio de Janeiro, Brazil), is only for screening when administered alone, despite its high sensitivity (99.5%) and specificity (99.0%).^14^ Therefore, any positive result from an oral fluid rapid test needs diagnostic confirmation through a finger puncture rapid test or another conventional test. To include the combination of two rapid tests – either two finger puncture rapid tests or one oral fluid test and one finger puncture test – in HIV diagnosis, national algorithms for HIV testing had to be updated.^15^ Moreover, legislation had to be reinterpreted through a ministerial decree so that the administration of oral fluid HIV rapid tests would no longer be restricted to health professionals, but open to any person trained in rapid testing by the health ministry.^16^

In partnerships with selected NGOs, the department ran a pilot project for the strategy between January 2014 and March 2015. The department selected 53 NGOs from all five geographical regions of Brazil through a bidding process that considered the NGOs’ experience in community work with key populations.

To support the implementation of the project, people from the health ministry held a two-day training session for NGO staff involved, either at the NGO’s office or at the local STI/AIDS and viral hepatitis coordination office. Staff were trained to implement the project by learning to develop an action plan; carry out the oral fluid test; counsel the person tested; inform only the patient of the results; make a referral to health services; and monitor the project.

The ministry provided NGOs with a booklet detailing the methods of the strategy and a training video showing how to do the tests.

When planning the interventions, NGOs and the local STI, AIDS and viral hepatitis coordinator mapped testing sites and coordinated referral services with local health management and municipal health facilities.

The NGO teams did all the work, from offering the test to referring those who tested positive to HIV services. The teams were free to develop their own ways of approaching people for testing. To improve uptake, the teams used their knowledge of each key population and their previous experience with community work. When possible, team members chose to approach people of the same population; for example, a transgender team member approached another transgender person. The teams carried out oral fluid testing at different times of the day in social venues they had mapped. The testing was free of charge and voluntary and the results were only revealed to the tested person. Although anyone could be tested at the venues, peers only offered tests to people they thought were part of the key populations. To be tested, the person had to fill out a consent form for taking the test and a registration form, which contained demographic information and information about the possible route of HIV transmission. Individual identification was not compulsory. Team members gave people with positive test results the address of a health facility where diagnosis could be confirmed and treatment and care provided. For those who chose not to provide personal identifiers on the registration form, the teams were unable to confirm enrolment in care.

The department developed a special monitoring and evaluation plan, a field log, monthly activity worksheets and technical reports with data collected during testing. Information from the registration form was inserted in SIMAV-pro, an online monitoring system that was developed specially for this strategy.

To ensure that those who tested positive got a confirmation and were referred to the local health services, a team from the department mediated NGOs’ contacts with local management and health services. The team also made frequent visits to the NGOs to ensure high-quality testing.

The results presented here are from a secondary analysis of data obtained anonymously during the implementation of the strategy, hence ethical approval of the study was not required.

## Relevant changes

During the pilot project, 29 723 oral fluid tests were administered; 791 (2.7%) were positive (Table 1). Transgender people had the highest proportion of positive results (10.7%; 172/1612), followed by men who declared themselves as commercial sex workers (8.7%; 165/1889) and men who have sex with men (4.8%; 292/6055). Of the 224 people who reported to have had at least one STI in the last 12 months before the test, 162 (7.2%) had a positive result.

**Table 1:** Characteristics of people tested for HIV with oral fluid rapid test, Brazil, January 2014 to March 2015

Characteristics	No. tested	No. with a positive result (%)	*P*^a^
**All**	29 723	791 (2.7)	–
**Age, years**			0.002
≤ 24	9 320	209 (2.2)	
25–34	9 776	259 (2.6)	
35–49	7 058	227 (3.2)	
≥ 50	3 190	82 (2.6)	
**Sex**			0.001
Female	13 085	205 (1.6)	
Male	16 546	583 (3.5)	
**Education**			0.021
Incomplete primary	4 856	156 (3.2)	
Complete primary	11 236	295 (2.6)	
High school or more	13 382	330 (2.5)	
**Population subgroup**			< 0.001
Transgender	1 612	172 (10.7)	
Men who have sex with men	6 055	292 (4.8)	
Heterosexual man	9 253	153 (1.7)	
Heterosexual woman	1 122	6 (0.5)	
Bisexual man	1 852	26 (1.4)	
Homosexual woman	9 618	140 (1.5)	
**Sex work**			< 0.001
No sex work	21 986	513 (2.3)	
Male sex worker	1 889	165 (8.7)	
Female sex worker	5 848	113 (1.9)	
**Use of psychoactive substances**			< 0.001
None	9 918	225 (2.3)	
One	13 554	354 (2.6)	
Two	3 661	123 (3.4)	
Three	1 757	50 (2.8)	
Four or more	833	39 (4.7)	
**Had at least one HIV test in lifetime**			< 0.001
Yes	13 760	240 (1.7)	
No	15 646	532 (3.4)	
**Had any STI during the last 12 months**			< 0.001
No	27 118	614 (2.3)	
Yes	2 244	162 (7.2)	

HIV: human immunodeficiency virus; STI: sexually transmitted infection.

^a^ To assess the differences in the proportions in each group, we conducted *χ*^2^ tests.

Note: Due to missing information, the sum of people in each sub-category does not add up to the total number of people tested.

Thirty-nine (4.7%) individuals of the 833 who reported use of at least four psychoactive substances (regardless of whether the substances were injectable or not) had positive results – twice as much as for those who did not report any use of psychoactive substances (2.3%; 225/9918; Table 1).

The proportion of positive results among people who were receiving their first HIV test (3.4%; 532/15 646) was twice the proportion observed among those who had already been tested at least once (1.7%; 240/13 760; Table 1).

The total cost of the pilot project was 643 882 United States dollars.

## Lessons learnt

A specific focus on increasing testing among key populations with the highest HIV burden^9^^,^^10^^,^^12^ resulted in an increase of early diagnoses. Two factors played a role in the increase. First, changes in the testing algorithm made it possible for trained peers to do the testing instead of health professionals. Second, the use of a non-invasive test for screening facilitated testing outside health-care centres.

The main lessons learnt are summarized in Box 1. Peer point-of-care using an oral fluid HIV test facilitated access for key populations to HIV testing and counselling. Furthermore, the people involved showed an increased acceptance of HIV testing, because the test was done by peers in convenient places and times of the day. The strategy also empowered the people working for participating NGOs by improving their ways of addressing key populations, increasing their knowledge about venues of social interest and tightening their relationship with the local health services. NGO team members developed skills and solved problems that moved the strategy forward.

Box 1 **Summary of main lessons learnt**

The strategy was designed to provide testing and prevention initiatives for populations with the highest human immunodeficiency virus (HIV) prevalence.

By reaching the key populations in their own environment, the peer-testing strategy increased HIV testing among people with higher HIV burden.

People accepted this testing strategy, in part because they were not exposed to possible discrimination experienced when visiting traditional health services.

The strategy promoted contacts between the Brazilian Federal Government and state and municipal STI and HIV/AIDS management. This kind of connection plays an important role within the operation of the Brazilian Unified Health System, since Brazil’s three government spheres – the union, states and municipalities – are mutually independent and their autonomy is ensured by the Brazilian Federal Constitution.

The proportion of positive tests identified during the project was almost seven times higher than in the general population – 2.7% versus 0.4%,^9^ respectively. This difference reinforces the need to expand initiatives focusing on the key populations to respond to the HIV epidemic in Brazil.
